# Phosphorylation of the Canonical Histone H2A Marks Foci of Damaged DNA in Malaria Parasites

**DOI:** 10.1128/mSphere.01131-20

**Published:** 2021-01-13

**Authors:** Manish Goyal, Adina Heinberg, Vera Mitesser, Sofiya Kandelis-Shalev, Brajesh Kumar Singh, Ron Dzikowski

**Affiliations:** a Department of Microbiology & Molecular Genetics, The Kuvin Center for the Study of Infectious and Tropical Diseases, IMRIC, The Hebrew University-Hadassah Medical School, Jerusalem, Israel; University at Buffalo

**Keywords:** malaria, *Plasmodium falciparum*, DNA damage, DNA repair, H2A phosphorylation, double-strand break

## Abstract

Plasmodium falciparum parasites proliferate within circulating red blood cells and are responsible for the deadliest form of human malaria. These parasites are exposed to numerous intrinsic and external sources that could cause DNA damage; therefore, they have evolved efficient mechanisms to protect their genome integrity and allow them to proliferate under such conditions. In higher eukaryotes, double-strand breaks rapidly lead to phosphorylation of the core histone variant H2A.X, which marks the site of damaged DNA. We show that in P. falciparum that lacks the H2A.X variant, the canonical P. falciparum H2A (PfH2A) is phosphorylated on serine 121 upon exposure to sources of DNA damage. We further demonstrate that phosphorylated PfH2A is recruited to foci of damaged chromatin shortly after exposure to sources of damage, while the nonphosphorylated PfH2A remains spread throughout the nucleoplasm. In addition, we found that PfH2A phosphorylation is dynamic and that over time, as the parasite activates the repair machinery, this phosphorylation is removed. Finally, we demonstrate that these phosphorylation dynamics could be used to establish a novel and direct DNA repair assay in P. falciparum.

**IMPORTANCE** Plasmodium falciparum is the deadliest human parasite that causes malaria when it reaches the bloodstream and begins proliferating inside red blood cells, where the parasites are particularly prone to DNA damage. The molecular mechanisms that allow these pathogens to maintain their genome integrity under such conditions are also the driving force for acquiring genome plasticity that enables them to create antigenic variation and become resistant to essentially all available drugs. However, mechanisms of DNA damage response and repair have not been extensively studied for these parasites. The paper addresses our recent discovery that P. falciparum that lacks the histone variant H2A.X phosphorylates its canonical core histone PfH2A in response to exposure to DNA damage. The process of DNA repair in *Plasmodium* was mostly studied indirectly. Our findings enabled us to establish a direct DNA repair assay for P. falciparum similar to assays that are widely used in model organisms.

## Abstract (precis)

Plasmodium falciparum is the deadliest human parasite that causes malaria when it reaches the bloodstream and begins proliferating inside red blood cells, where the parasites are particularly prone to DNA damage. The molecular mechanisms that allow these pathogens to maintain their genome integrity under such conditions are also the driving force for acquiring genome plasticity that enables them to create antigenic variation and become resistant to essentially all available drugs.

## INTRODUCTION

Plasmodium falciparum is the protozoan parasite responsible for the deadliest form of human malaria. This parasite is estimated to infect 200 million to 300 million people worldwide each year, resulting in approximately half a million deaths, primarily of young children (1). P. falciparum replicates within the circulating red blood cells (RBCs) of an infected individual, and its virulence is attributed to its ability to modify the erythrocyte surface and to evade the host immune attack. During their intraerythrocytic development, *Plasmodium* parasites replicate their haploid genomes multiple times through consecutive mitosis cycles called schizogony, which makes them particularly prone to errors during DNA replication. In addition, blood-stage parasites that live in a highly oxygenated environment produce potent DNA-damaging agents while digesting hemoglobin and are exposed to oxidative substances released from immune cells (2).

Therefore, *Plasmodium* parasites that are exposed to numerous sources that can damage their DNA must have evolved efficient mechanisms to protect their genome integrity. Orthologues to many of the proteins involved in the DNA damage response (DDR) are encoded in P. falciparum genome (2), including those involved in homologous recombination (HR), microhomology-mediated end joining (MMEJ) (2), and mismatch repair machineries (3). However, these mechanisms have not been extensively studied for these parasites. It appears that malaria parasites utilize both HR and an alternative end joining pathway to maintain their genome integrity (4). Thus, in the absence of a homologous sequence in their haploid genome that can serve as a template for HR, blood-stage parasites primarily repair double-strand breaks (DSB) using the alternative microhomology-mediated end joining mechanism (4, 5).

In mammals, a single double-strand break of the DNA triggers the DDR that rapidly leads to extensive ataxia telangiectasia mutated (ATM) kinase-dependent phosphorylation of the core histone isoform H2A.X to form phospho-H2A.X (γ-H2A.X), which marks the site of damaged DNA (6). However, the P. falciparum genome lacks an orthologue of the H2A.X variant, and it encodes only two H2A variants, the canonical P. falciparum H2A (PfH2A; PF3D7_0617800) and PfH2A.Z (PF3D7_0320900), which was shown to be associated with a subset of active promoters (7). Previous histone phosphorylation analysis suggested that PfH2A could be phosphorylated on serine 121 (8). We were interested to determine whether in P. falciparum phosphorylation of PfH2A might be correlated with DNA damage. We show that these parasites phosphorylate the canonical PfH2A on serine 121 in response to DNA damage and that the phosphorylated PfH2A is recruited to the damaged foci. In addition, the ability to specifically detect the dynamics of this phosphorylation using an anti-γ-H2A.X antibody provides a useful marker for studying DNA damage mechanisms which allowed us to establish a direct DNA repair assay in P. falciparum.

## RESULTS

**FIG 1:**
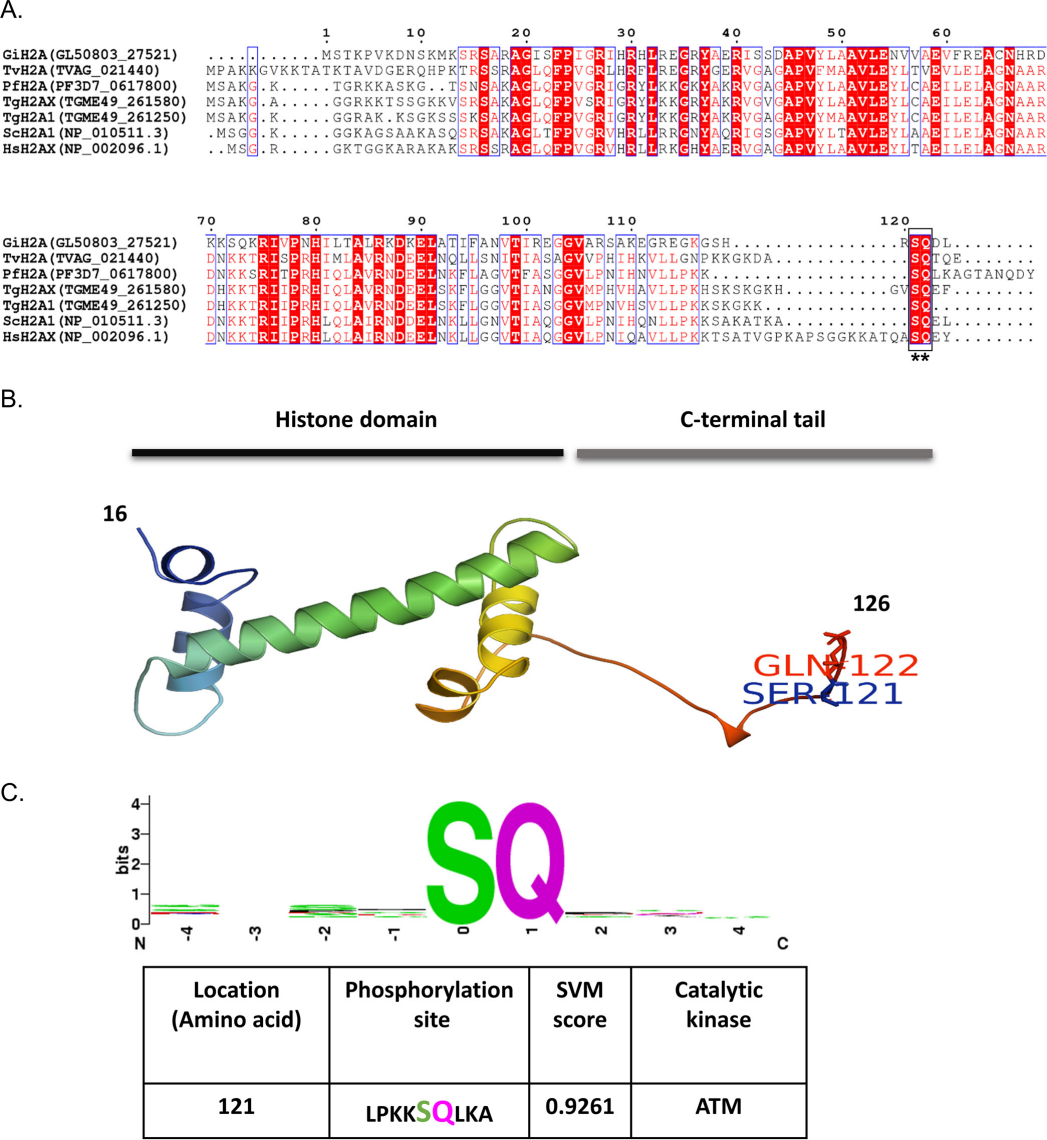
*In silico* analyses of putative ATM kinase-specific phosphorylation site (conserved SQ motif) in PfH2A. (A) Multiple-sequence alignment of amino acid sequences of PfH2A and some of H2A variants from human, budding yeast, and protozoan parasites using ESPript 3. Similar and identical amino acids are boxed and marked with a red background. The conserved C′-terminal SQ motif is underlined with asterisks. Database accession numbers or gene names appear in parentheses. Abbreviations: PfH2A, P. falciparum histone H2A; HsH2AX, Homo sapiens histone H2AX; ScH2A1, Saccharomyces cerevisiae histone H2A1; TgH2A1, Toxoplasma gondii histone H2A1; TgH2AX, Toxoplasma gondii histone H2A.X; TvH2A, Trichomonas vaginalis histone H2A; GiH2A, Giardia intestinalis histone H2A. (B) 3D homology model of PfH2A (developed using structure homology-modeling server SWISS-MODEL) showing the core histone domain and that the extended C-terminal tail contains the conserved S^121^Q^122^ motif. (C). *In silico* prediction of ATM kinase-specific phosphorylation sites in PfH2A (using KinasePhos, version 2.0). The sequence-based amino acid coupling-pattern analysis and solvent accessibility of PfH2A suggest serine 121 as the most prominent ATM kinase-specific phosphorylation site.

In model systems, phosphorylation of H2A.X is elevated following exposure to DNA-damaging agents and is commonly used as a marker for double-strand breaks (6). The phosphorylation of serine 139 found on a conserved SQ (serine-glutamine) motif of mammalian H2A.X serves as a differential epitope for detection of the phosphorylated form, known as γ-H2A.X. *Plasmodium* parasites do not contain a gene encoding the H2A.X variant in their genome, but instead they express the canonical H2A and the H2A.Z variants (PF3D7_0617800 and PF3D7_0320900, respectively). In the absence of a good marker for DNA damage in *Plasmodium*, we were interested to test whether the antibody that recognizes γ-H2A.X in mammals could be used as a marker for DNA damage in P. falciparum. As a first step, we aligned the two *Plasmodium* H2A variants with human H2A.X and noted that only the canonical PfH2A has a long C-terminal tail containing the SQ motif, which is conserved among *Plasmodium* species, while no SQ motif was found in PfH2A.Z (see Fig. S1 in the supplemental material). This SQ motif is conserved among the canonical H2A proteins of several protozoan parasites, such as P. falciparum, Giardia lamblia, and Trichomonas vaginalis, that lack H2A.X orthologues. Similarly, the budding yeast Saccharomyces cerevisiae lacks the H2A.X orthologue, and instead, its canonical H2A was found to be phosphorylated on serine 129 (SQ motif). This phosphorylation is detected by the anti-γ-H2A.X antibody, and thus, the phosphorylated form of S. cerevisiae H2A is often referred to as γ-H2A.X (9). Interestingly, in contrast to *Plasmodium* spp., the apicomplexan parasite Toxoplasma gondii has an H2A.X variant (TgH2A.X) in addition to its canonical H2A (Fig. 1A). As expected, *in silico* structural prediction of PfH2A suggests that the SQ motif is found in its C′-terminal tail (Fig. 1B) and that this motif is likely to be an ATM kinase phosphorylation site (Fig. 1C), similar to the serine 139 of the mammalian H2A.X, which is the major residue phosphorylated in response to DNA damage by the ATM kinase (6).

10.1128/mSphere.01131-20.1 FIG S1 (A) Multiple-sequence alignment of amino acid sequences of histone H2A variants of P. falciparum and human H2A.X, indicating that PfH2A.Z does not contain an SQ motif in its C′ tail. (B) Multiple-sequence alignment of amino acid sequences of histone H2A from different *Plasmodium* spp., indicating that the SQ motif in the C′ tail is highly conserved. The similar and identical amino acids are boxed and marked with a red background, respectively. The conserved C-terminal SQ motif is marked with an asterisk. Abbreviations (corresponding accession numbers in https://plasmodb.org): PfH2A, Plasmodium falciparum histone H2A (PF3D7_0617800); PfH2AZ, Plasmodium falciparum histone H2AZ (PF3D7_0320900); HsH2AX, Homo sapiens histone H2A (NP_002096.1); PvxH2A, Plasmodium vivax histone H2A (PVX_114015); PvH2A, Plasmodium vinckei histone H2A (YYE_02539); PyH2A, *Plasmodium yoelli* histone H2A (PY05076); PkH2A, Plasmodium knowlesi histone H2A (PKNH_1132600); PcH2A, Plasmodium chabaudi histone H2A (PCHAS_1116500); PbH2A, Plasmodium berghei histone H2A (PBANKA_1117000). Download FIG S1, TIF file, 0.2 MB. Copyright © 2021 Goyal et al. 2021 Goyal et al. This content is distributed under the terms of the Creative Commons Attribution 4.0 International license.

**FIG 2:**
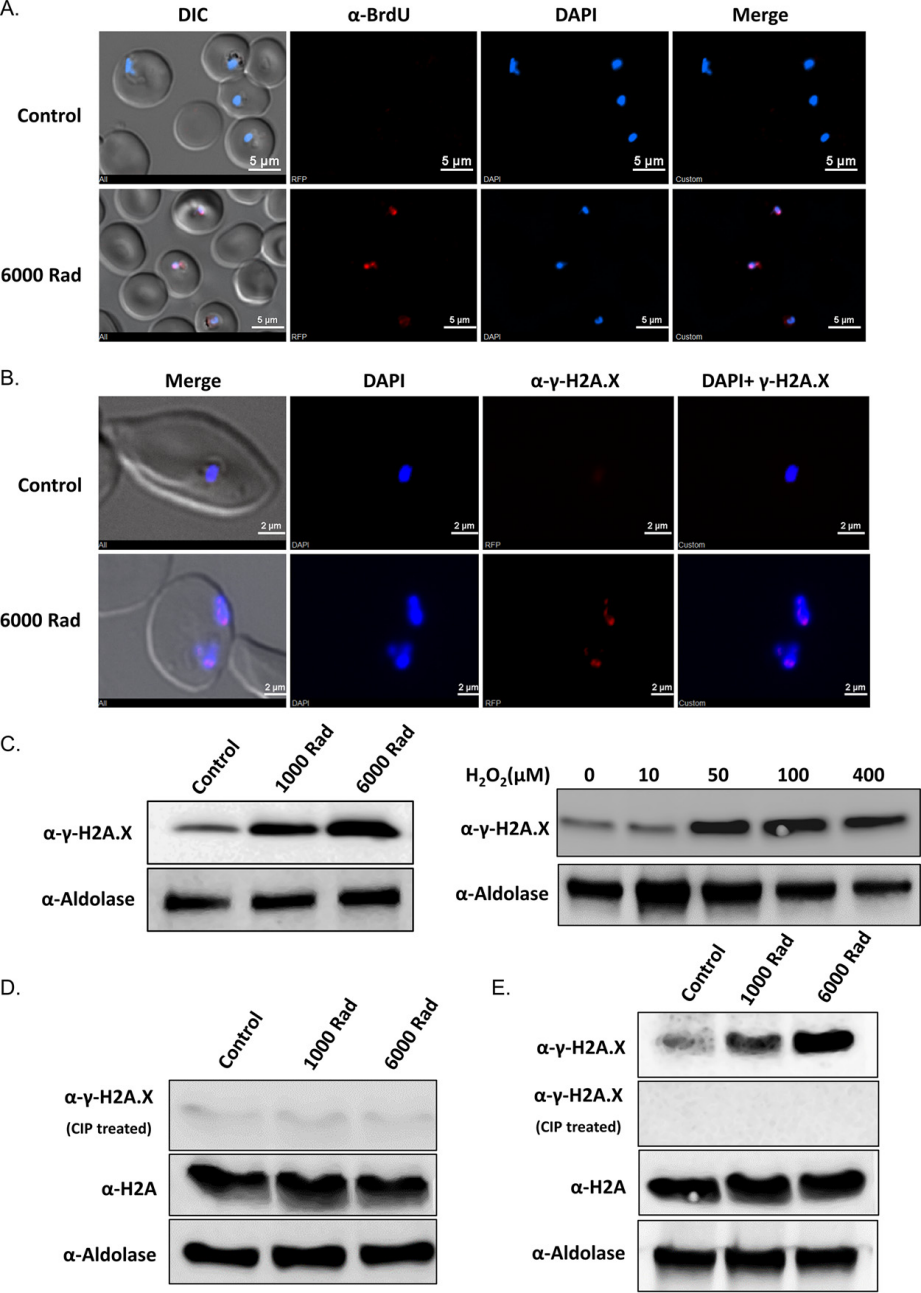
DNA damage in P. falciparum causes histone H2A phosphorylation. (A) DNA fragmentation imaging by TUNEL assay of RBCs infected with NF54 P. falciparum parasites exposed to X-ray irradiation (6,000 rads) showing nuclear foci of damaged DNA. (B) Immunofluorescence analysis of X-ray-irradiated parasites (6,000 rads), using anti-γ-H2A.X (S^P^Q) antibody, shows foci of phosphorylation signal in the nucleus. (C) Western blot analysis, using anti-γ-H2A.X(S^P^Q) antibody, of protein extracts from parasites exposed to increasing levels of X-ray irradiation (left) and from parasites treated with increasing concentrations of H_2_O_2_ (right). (D and E). The anti-γ-H2A.X (S^P^Q) antibody specifically recognizes phosphorylated PfH2A and does not cross-react with nonphosphorylated PfH2A. Proteins from parasites, which were irradiated with increasing doses of X-ray radiation (control [no irradiation] and 1,000 and 6,000 rads), were subjected to Western blot analysis using either anti-γ-H2A.X (S^P^Q) antibody or anti-H2A antibody. The membrane was either incubated with calf intestine phosphatase (CIP) before incubation with the antibodies (D) or incubated with the antibodies, stripped, treated with CIP, and reincubated with the antibodies (E). Anti-aldolase antibody was used as a loading control. The anti-γ-H2A.X (S^P^Q) antibody detected increasing levels of protein associated with the increasing levels of irradiation only without CIP treatments, while the anti-H2A antibody detected constant protein levels even after CIP treatment.

To test if PfH2A is indeed phosphorylated in response to exposure of the parasite to a source of DNA damage, we exposed tightly synchronized ring-stage NF54 parasites to X-ray irradiation. We chose to irradiate early-stage parasites that do not replicate their DNA and do not have hemozoin, and therefore, the detected DNA damage should be mostly due to the exogenous source. We used terminal deoxynucleotidyltransferase-mediated dUTP-biotin nick end labeling (TUNEL) assays as direct evidence that exposure of the parasite to 6,000 rads caused DNA damage, which was detected in most parasites’ nuclei (Fig. 2A). We then used the γ-H2A.X antibody for immunofluorescence assays (IFA) and were able to detect strong signals within the parasites’ nuclei after exposure to X-ray irradiation (Fig. 2B). This observation was further confirmed using γ-H2A.X antibody on proteins extracted from parasites exposed to increasing levels of irradiation, which showed a corresponding elevation in the levels of γ-PfH2A (Fig. 2C, left, and Fig. S2). Similarly, exposing the parasites to hydrogen peroxide (H_2_O_2_), another source of DNA damage, caused an increase in the levels of γ-PfH2A recognition (Fig. 2C, right). To ensure that the anti-γ-H2A.X antibody specifically recognized the phosphorylated form of PfH2A and did not cross-react with the nonphosphorylated form, we incubated the extracted proteins with calf intestine phosphatase (CIP), which removes phosphate residues. The CIP treatment specifically abolished immunoblot detection using the anti-γ-H2A.X antibody, while the nonphosphorylated PfH2A was detected at similar levels in parasites exposed to increasing X-ray levels (Fig. 2D). In addition, when we initially probed with the anti-γ-H2A.X antibody after irradiation, we observed increasing levels of phosphorylation; however, when the blot was stripped, treated with CIP, and reprobed, the anti-γ-H2A.X antibody signal disappeared, while detection of the canonical PfH2A was unchanged (Fig. 2E). Together, these data suggest that PfH2A is phosphorylated in response to DNA damage and that the anti-γ-H2A.X antibody is specific to the phosphorylated form of PfH2A.

10.1128/mSphere.01131-20.2 FIG S2 Western blot analysis showing the entire gel of protein extracted from parasites before (control) and after exposure to DNA X-ray irradiation (6,000 rads). Detection using the anti-γ-H2A.X(S^P^Q) antibody indicated an increase in the level of PfH2A phosphorylation following irradiation, while no additional bands were observed. Download FIG S2, TIF file, 1.6 MB. Copyright © 2021 Goyal et al. 2021 Goyal et al. This content is distributed under the terms of the Creative Commons Attribution 4.0 International license.

**FIG 3:**
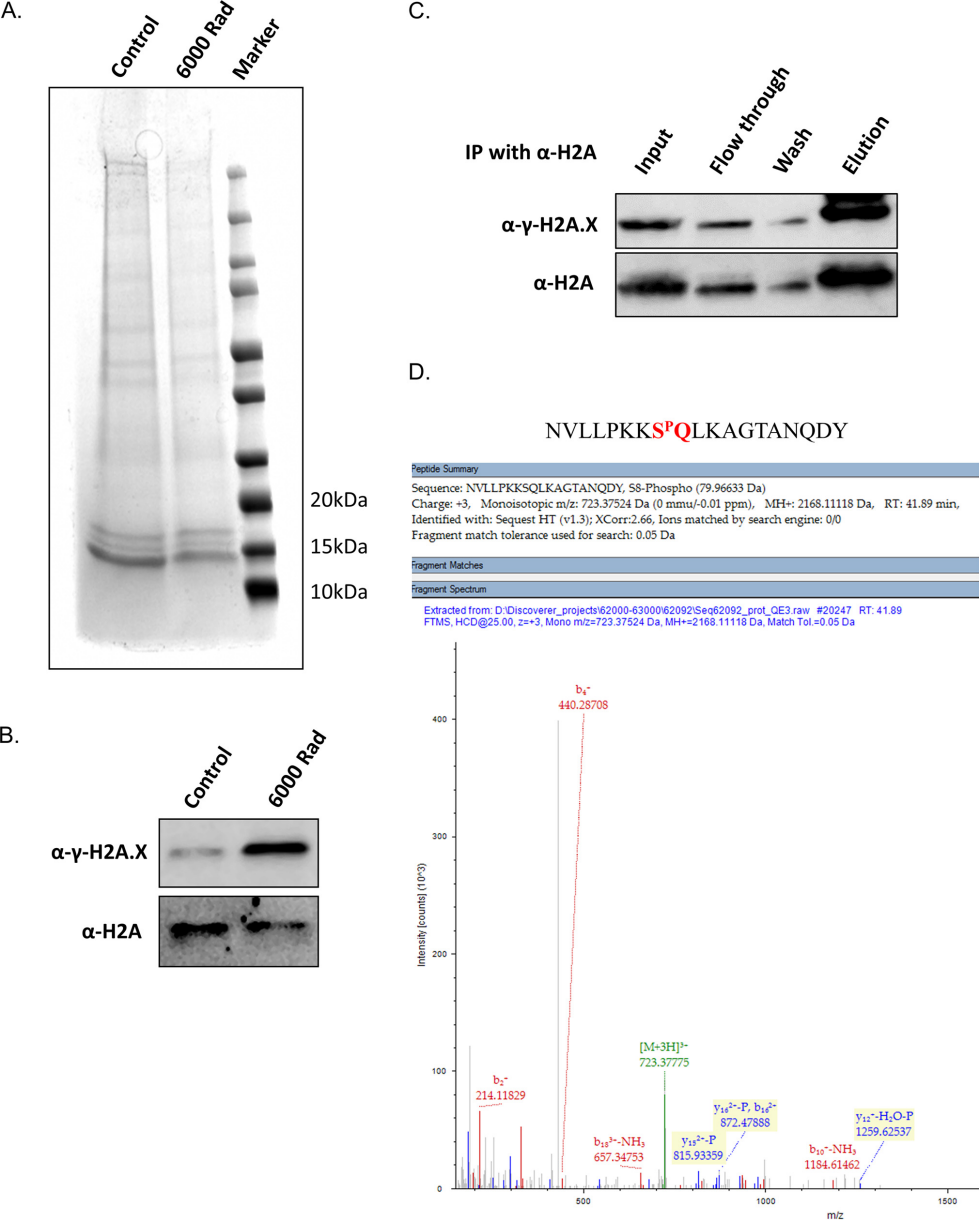
Histone extraction followed by mass spectrometry shows that serine 121 of PfH2A is phosphorylated upon exposure to X-ray irradiation. (A) SDS-PAGE analysis of histone extraction and purification from X-ray-irradiated (6,000 rads) and untreated parasites. (B) Western blot analysis of total histone extracted from untreated and X-ray-treated (6,000 rads) parasites using anti-γ-histone H2AX (S^P^Q) and anti-PfH2A antibodies. (C) X-ray irradiated parasites (6,000 rads) were subjected to immunoprecipitation (IP), using anti-H2A antibody, followed by Western blot analysis using both anti-γ-H2A.X (S^P^Q) and anti-H2A antibodies. (D) Trypsin digestion followed by mass spectrometry analysis identified phosphorylation of serine 121 of PfH2A in the irradiated parasites.

To further confirm that the phosphorylation detected by the anti-γ-H2A.X antibody, following parasite exposure to DNA damage, is indeed phosphorylation of the canonical PfH2A, we extracted and purified histones from parasites that were either exposed or not exposed to X-ray irradiation. Immunoblot analysis of the total histone extract showed an increase in the level of the phosphorylated form of PfH2A following irradiation, while the total levels of PfH2A were similar (Fig. 3A and B). We further exposed parasites to X-ray irradiation and performed immunoprecipitation (IP) of total PfH2A using an anti-H2A antibody. The IP fractions were subjected to immunoblotting with the anti-γ-H2A.X antibody, which demonstrated significant enrichment of the phosphorylated form of PfH2A in the elution (Fig. 3C). This fraction was subjected to trypsin digestion followed by mass spectrometry analysis, which identified phosphorylation on serine 121 of PfH2A (Fig. 3D).

**FIG 4:**
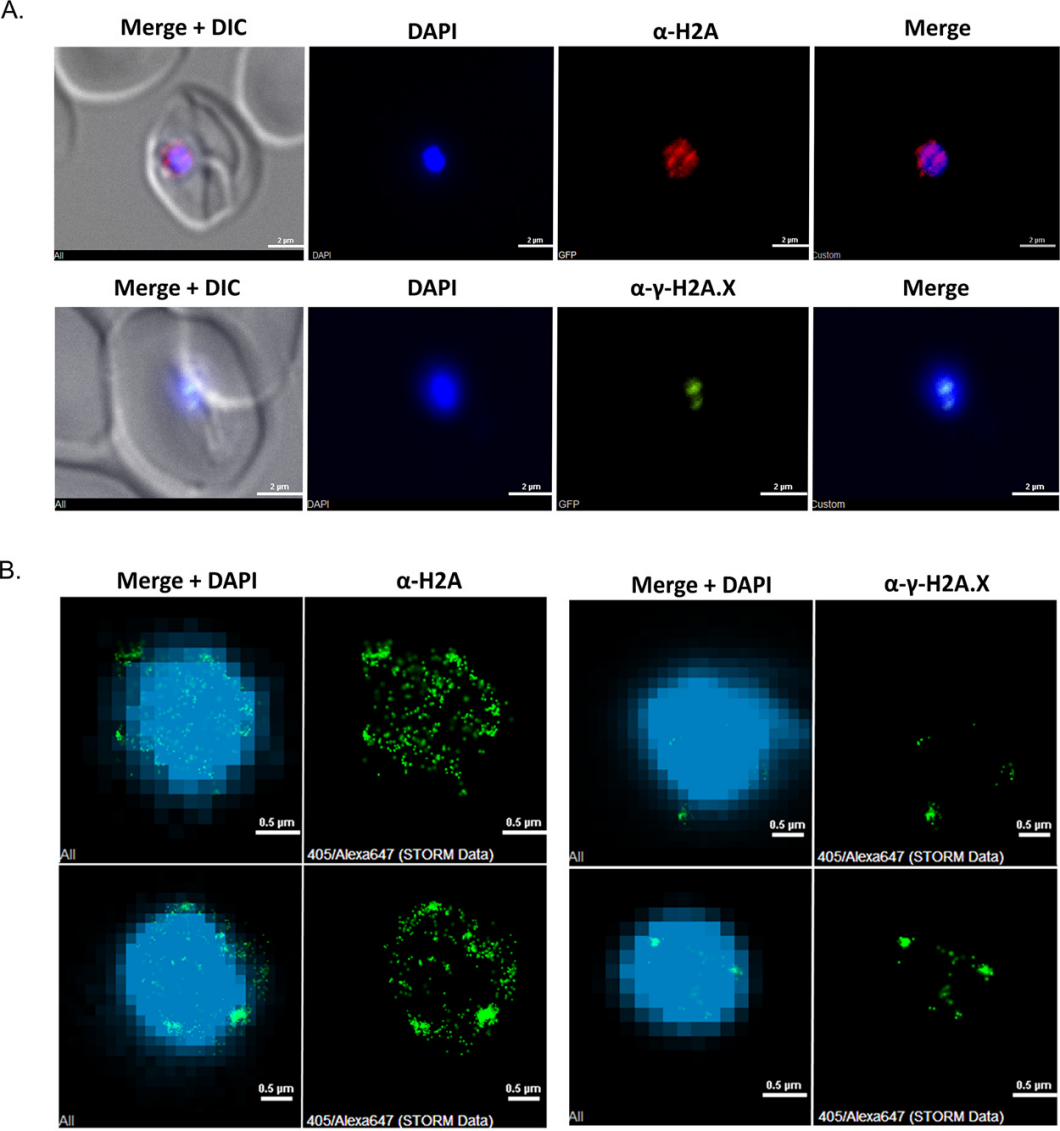
Phosphorylated PfH2A is located at distinct nuclear foci, while the nonphosphorylated PfH2A is spread throughout the nucleoplasm. Immunofluorescence of PfH2A and phosphorylated PfH2A (A; red, anti-γ-H2A; green, anti-γ-H2A.X; blue, DAPI; scale bar, 2 μm) and superresolution STORM imaging of PfH2A and phosphorylated PfH2A (B; green, Alexa Fluor 647 staining each of the PfH2A isoforms; blue, YOYO1 staining of DNA at low resolution for orientation; scale bar, 0.5 μm) in P. falciparum nuclei following X-ray irradiation (6,000 rads).

The specificity of the anti-γ-H2A.X antibody to the phosphorylated form of PfH2A allowed us to image its nuclear distribution compared with that of the nonphosphorylated PfH2A in irradiated parasites. Immunofluorescence assay using anti-H2A and anti-γ-H2A.X antibodies indicated that while the canonical PfH2A is spread throughout the nucleoplasm, its phosphorylated form is found at distinct foci (Fig. 4A). To further validate this observation, we performed superresolution stochastic optical reconstruction microscopy (STORM) imaging, which enabled us to image the nuclear distribution of the two forms of PfH2A in detail at the nanoscale level. This analysis clearly demonstrated the differential distribution of the two PfH2A forms in the nucleoplasm. The nonphosphorylated PfH2A is indeed spread throughout the nucleoplasm, while the phosphorylated form is much less abundant and is found at distinct nuclear foci (Fig. 4B).

**FIG 5:**
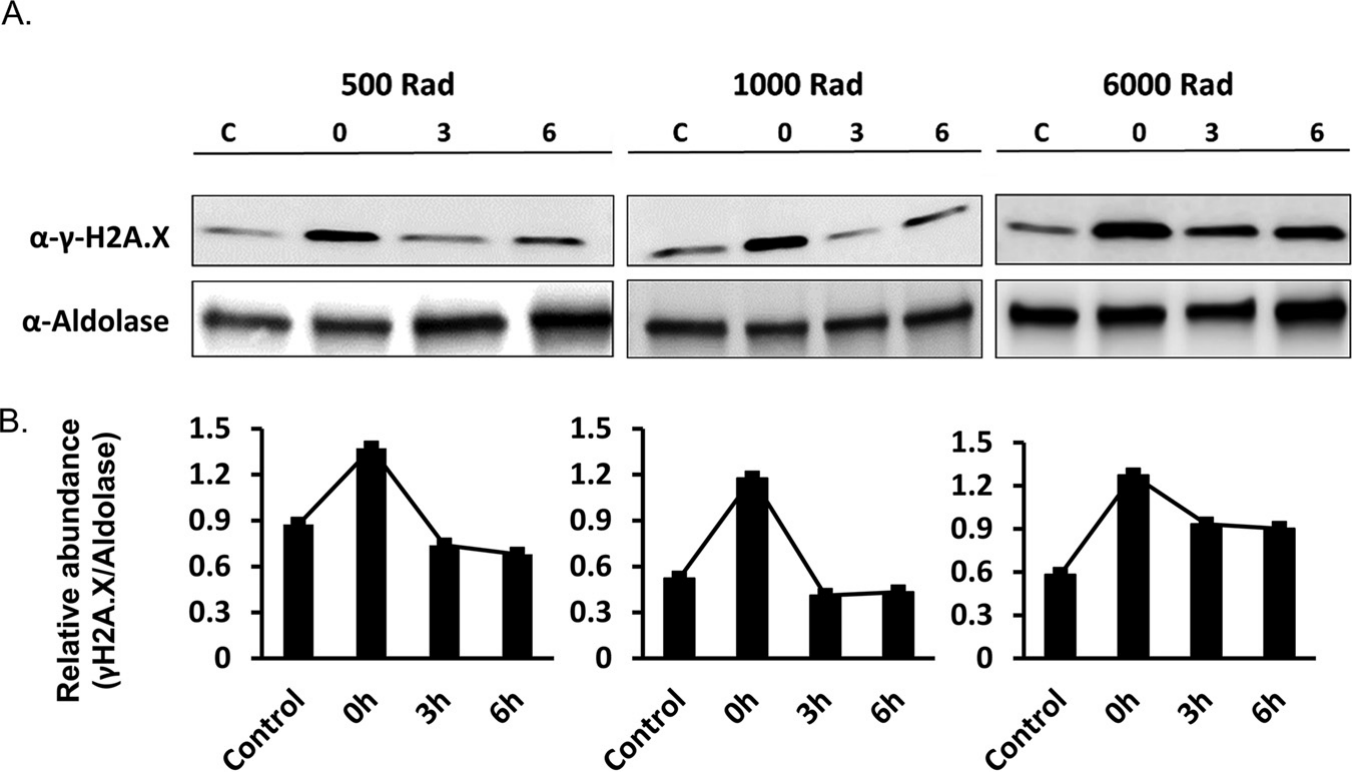
DNA damage and repair assay in P. falciparum infected RBCs (iRBCs). (A) Parasites were treated with different doses of X-ray radiation (i.e., 500, 1,000, and 6,000 rads) and put back in culture (3 h and 6 h) to allow them to repair their damaged DNA. Protein extracts from these parasites were then used for Western blot analysis with anti-γ-H2A.X (S^P^Q) antibody and anti-aldolase antibody as a loading control. The ability of the parasites to repair their damaged DNA is demonstrated by the rapid reduction in the levels of phosphorylated PfH2A found 3 h after irradiation. Lanes C, parasites that were not exposed to irradiation and used as control. (B) Semiquantitative densitometry analysis of the Western blot presented in panel A. Shown is quantification of the changes in the ratio between the signal detected by the anti-γ-H2A.X and anti-aldolase antibodies before irradiation, immediately after irradiation, and 3 h and 6 h after irradiation. The density of each band is presented as a proportion of the total signal obtained.

Thus far, the process of DNA repair in *Plasmodium* was mostly studied indirectly by measuring the recovery of parasites in culture after exposure to a source of DNA damage (10). In addition, repair mechanisms were studied directly by creating a transgenic inducible DSB system by integrating an *I-SceI* cleavage site into the P. falciparum genome and sequencing of the repaired locus after induction of the *I-SceI* endonuclease (4). Our data strongly suggest that phosphorylation of PfH2A could be used as a specific and immediate marker for damaged DNA in P. falciparum. Therefore, we were interested in examining the dynamics of this phosphorylation over time after exposure to X-ray irradiation, hypothesizing that it could be exploited to establish a direct DNA repair assay for P. falciparum similar to assays that are widely used in model organisms. We exposed parasite cultures to different levels of X-ray irradiation and measured the levels of PfH2A phosphorylation over time. We observed that the levels of PfH2A phosphorylation, which had increased immediately after irradiation, decreased already 3 h after irradiation to levels that were similar to those prior to irradiation (Fig. 5). These data imply that during this period, the parasites were able to activate their DNA repair machinery, and thus, these dynamics could be used as a valuable tool to study DNA repair in malaria parasites.

## DISCUSSION

In any living organism, the ability to repair damaged DNA is key for maintaining genome integrity. This DNA damage repair (DDR) machinery should be extremely efficient in organisms, such as *Plasmodium* parasites, that are continuously exposed to numerous intrinsic and exogenous sources that may damage their DNA. In addition to its crucial role for the parasites’ basic biological functions under these conditions, efficient DDR machinery contributes to the parasites’ ability to expand their antigenic repertoire and to maintain mutations that enable them to resist drug treatment. However, although many regulators of DDR were identified encoded in the *Plasmodium* genome, the mechanisms for DDR in these parasites remained understudied and poorly understood. A major obstacle for advancing our knowledge on DDR machinery in *Plasmodium* is the lack of good molecular markers for damaged DNA and the inability to perform an accurate assay that directly measures the kinetics of DNA repair. Here, we show that exposure of P. falciparum parasites to X-ray irradiation and H_2_O_2_, which cause double-strand breaks (DSB), leads to increased phosphorylation of the canonical PfH2A. We found that although *Plasmodium* has no H2A.X variant, the canonical PfH2A is phosphorylated on the SQ motif found in its C′-terminal tail and that the phosphorylated PfH2A could be differentiated from the nonphosphorylated form of this core histone protein. Since PfH2A is phosphorylated following exposure to DNA-damaging agents, the quantitative measurement of phosphorylated PfH2A can act as a sensitive molecular marker for DNA damage in P. falciparum. Most of the approaches employed to date to study DNA damage in *Plasmodium* rely on measuring the relative instability of DNA damage products under alkaline conditions (comet assay) (11, 12) or on the relative expression of DNA damage and repair genes (reverse transcription-quantitative PCR [qRT-PCR]) (11, 13, 14). In addition, thus far, the ability of P. falciparum parasites to repair DNA damage has been estimated by the rate of recovery of parasite populations exposed to DNA-damaging agents, i.e., the time it takes for these populations to reach approximately 5% parasitemia (usually 10 to 20 days) (10). Any delay in recovery was then interpreted as a malfunction of the repair machinery, which is, of course, indirect evidence reflected 2 weeks after the actual repair has happened. In this manner, the analysis of PfH2A phosphorylation kinetics can fulfill the need for a direct, simple, sensitive/quantitative, and reproducible way of measuring DNA damage and repair kinetics in *Plasmodium* in a timescale of minutes to hours, which better represents the velocity of the repair machinery.

In higher organisms, phosphorylation of histone variant H2A.X is a highly specific and sensitive molecular marker for monitoring DNA damage and repair (15, 16). However, in some organisms, other histone H2A variants undergo phosphorylation in response to exposure to DNA damage. For example, in Drosophila melanogaster, H2A.Z is phosphorylated in response to DNA damage instead of H2A.X (17), and in the budding yeast Saccharomyces cerevisiae, which does not encode an H2A.X variant, the canonical H2A is phosphorylated at the serine found near its C terminus at an SQ motif (18), similar to what we report here for P. falciparum. This also appears to be the case in protozoan species in which histone H2A.X is either missing or replaced by other histone variants. A marked example is in Trypanosoma brucei and other trypanosomatids as well, in which histone H2A undergoes phosphorylation at a threonine residue (Thr 130) instead of serine in response to DNA damage (19). Interestingly, in the apicomplexan parasite Toxoplasma gondii, which does contain an H2A.X variant, the canonical H2A (also named H2A1) was also proposed to be phosphorylated at a C-terminal SQ motif as a response to DSB (20). This may suggest the possibility of functional redundancy among these variants that could be exploited through evolution for functional replacement by the canonical H2A when the H2A.X variant is missing. This is somehow supported by the high level of conservation of the SQ motif in the canonical H2A of other protozoan and in particular in other *Plasmodium* species that face similar exposure to sources of DNA damage, such as P. falciparum. Interestingly, lower eukaryotes prefer high-fidelity HR as the mechanisms to repair DSB, while higher eukaryotes prefer nonhomologous DNA end joining (NHEJ) (21). We noted that both P. falciparum and S. cerevisiae, which phosphorylate their canonical H2A proteins in response to DSB, use HR for repair, while T. gondii, which encodes and phosphorylates its H2A.X variant, uses mainly NHEJ, similar to higher eukaryotes (21, 22). One can speculate whether the preference for NHEJ might have evolved in association with the H2A.X variant.

In higher eukaryotes, histone H2A.X is known to be phosphorylated by members of phosphatidylinositol 3-kinase family (PI3K) namely, ataxia telangiectasia mutated (ATM) kinase, ATM Rad-3-related kinase (ATR), and DNA-dependent protein kinase (DNA-PK) (23). However, to the best of our knowledge, only one PI3K was identified in *Plasmodium* (24), while other members of this family are not well characterized. Interestingly, in the apicomplexan parasite T. gondii, an ATM kinase orthologue (TGME49_248530) was proposed to be involved in TgH2A.X phosphorylation (25). Incubation of cultured parasites with a known ATM kinase inhibitor (KU-55933), which was tested as a potential anti-T. gondii agent, caused cell cycle arrest and was able to inhibit phosphorylation of TgH2A.X (26). The single PI3K which was previously identified in P. falciparum (PF3D7_0515300) shows some sequence conservation with T. gondii (TGME49_248530) and human (NCBI:protein database accession number AAB65827) ATM kinases. However, this P. falciparum PI3K (PF3D7_0515300), which was shown to be important for hemoglobin digestion, was localized to the parasite plasma membrane (PM), parasitophorous vacuole membrane (PVM), and food vacuole (FV) but did not appear to be localized to the nucleus (24). Thus, the *Plasmodium* ATM kinase homologue that phosphorylates PfH2A has yet to be identified.

Overall, the identification of PfH2A phosphorylation as a marker for DNA damage and the ability to quantify and time the appearance and disappearance of this marker in response to exposure to sources of DNA damage open new possibilities for understanding the mechanisms of DNA damage and repair that contribute to the persistence and pathogenicity of these important pathogens.

## MATERIALS AND METHODS

### Parasite culture.

All experiments were conducted on the human malaria NF54 parasite line. The parasites were cultivated at 37°C in an atmosphere of 5% oxygen, 5% carbon dioxide, and 90% nitrogen at 5% hematocrit in RPMI 1640 medium, 0.5% Albumax II (Invitrogen), 0.25% sodium bicarbonate, and 0.1 mg/ml of gentamicin. The parasites were synchronized using Percoll/sorbitol gradient method in which infected RBCs were layered on a step gradient of 40/70% Percoll containing 6% sorbitol. The gradient was subsequently centrifuged at 12,000 × *g* for 20 min at room temperature. The late-stage synchronized parasites were recovered from the interphase, washed twice with complete culture medium, and placed back in culture. The percent parasitemia was calculated by SYBR green I DNA stain (Life Technologies) using a CytoFLEX (Beckman Coulter) flow cytometer.

### Bioinformatics analyses.

The full-length sequences of histone H2A and its variants were obtained from different species based on sequence similarity. Multiple-sequence alignment of PfH2A (PF3D7_0617800) and other histone H2A variants from different species was performed using CLUSTALW and further analyzed using ESPript3 program. A homology model of the PfH2A was built by comparative modeling using the crystal structure of histone H2A (Protein Data Bank entry 1EQZ, chain A) by using the SWISS-MODEL server. The structure visualization of the PfH2A three-dimensional (3D) model was performed using the PyMOL program.

### DNA damage of parasites by X-ray irradiation and H_2_O_2_.

DNA damage in the parasites was performed by X-ray irradiation using a PXi precision X-ray irradiator set at 225 kV and 13.28 mA. In brief, 2% ring-stage NF54 parasites were exposed to different doses of X-ray irradiation (1,000, 3,000, and 6,000 rads). After irradiation, parasites were either collected immediately (i.e., 15 min after irradiation) or put back in culture with fresh medium for further analysis at different time points as mentioned elsewhere. The level of DNA damage was measured by *in situ* DNA fragmentation (TUNEL) assay and phosphorylated H2A by Western blotting as described above. To check the hydrogen peroxide (H_2_O_2_)-mediated DNA damage, ring-stage-infected RBCs (∼2%) were treated with different concentrations of H_2_O_2_ (0 to 10, 50, 100, and 400 μM) for 1 h at 37°C. Parasites were collected from RBCs by saponin lysis, and the level of DNA damage was measured by phosphorylated H2A following Western blotting as described above.

### *In situ* DNA fragmentation (TUNEL) assay.

Tightly synchronized ring-stage parasites (NF54) were fixed for 30 min in freshly prepared fixative (4% paraformaldehyde and 0.005% glutaraldehyde). After fixation, cells were rinsed three times with phosphate-buffered saline (PBS) and incubated with permeabilization solution (0.1% Triton X-100 in PBS) for 10 min on ice. The cells were washed twice with PBS and one time with wash buffer supplied with TUNEL assay kit bromodeoxyuridine (BrdU) red (Abcam; catalogue number ab6610). TUNEL assay was performed as per manufacturer guidelines. Briefly, following a washing, 50 μl of TUNEL reaction mixture (DNA labeling solution) was added to each sample. The cells were incubated for 60 min at 37°C with intermittent shaking. The cells were then washed three times with rinse buffer (5 min each time) and resuspended in 100 μl of antibody solution for 30 min at room temperature. The cells were then washed three times with PBS, mounted using Invitrogen Molecular Probes ProLong Gold antifade reagent with 4′,6-diamidino-2-phenylindole (DAPI), and imaged using a Nikon Eclipse Ti-E microscope equipped with a CoolSNAPMyo charge-coupled-device (CCD) camera.

### Western blotting.

Infected RBCs were lysed with saponin, and parasites were pelleted down by centrifugation. The parasite pellet was subsequently washed twice with PBS and lysed in 2× Laemmli sample buffer. The protein lysates were centrifuged and the supernatants were subjected to SDS-PAGE (gradient, 4 to 20%; Bio-Rad) and electroblotted onto a nitrocellulose membrane. Immunodetection was carried out by using rabbit anti-γ-H2A.X (S^P^Q) primary antibody (generated using a peptide containing the S^P^Q motif [catalogue number 9718S, 1:1,000; Cell Signaling]), anti-H2A antibody (Abcam; catalogue number ab88770, 1:1,000), and rabbit polyclonal anti-aldolase antibody (1:3,000) (27). The secondary antibodies used were antibodies conjugated to horseradish peroxidase (HRP), goat anti-rabbit (Jackson ImmunoResearch Laboratories; 1:10,000). The immunoblots were developed in EZ/ECL solution (Israel Biological Industries).

### Immunofluorescence assay.

Immunofluorescence assay (IFA) was performed as described previously, with minor modifications (28). In brief, iRBCs were washed twice with PBS and resuspended in a freshly prepared fixative solution (4% paraformaldehyde [Electron Microscopy Sciences] and 0.0075% glutaraldehyde [Electron Microscopy Sciences] in PBS) for 30 min at room temperature. Following fixation, iRBCs were permeabilized with 0.1% Triton X-100 (Sigma) in PBS and then blocked with 3% bovine serum albumin (BSA; Sigma) in PBS. Cells were then incubated with primary rabbit anti-γ-H2A.X (S^P^Q) (Cell Signaling; catalogue number 9718S, 1:300) and anti-H2A (Abcam; catalogue number ab8870, 1:100) antibodies for 1.5 h at room temperature and washed three times in PBS. Following this, cells were incubated with Alexa Fluor 488 goat anti-rabbit (Life Technologies; 1:500) or Alexa Fluor 568 goat anti-rabbit (Life Technologies; 1:500) antibodies for 1 h at room temperature. Cells were washed three times in PBS and laid on polytetrafluoroethylene (PTFE) printed slides (Electron Microscopy Sciences) and mounted in ProLong Gold antifade reagent with DAPI (Molecular Probes). Fluorescent images were obtained using a Plan Apo λ 100× oil immersion lens (numerical aperture [NA] = 1.5; working distance [WD] = 130 μm) on a Nikon Eclipse Ti-E microscope equipped with a CoolSNAPMyo CCD camera. Images were processed using NIS-Elements AR (4.40 version) software.

### STORM imaging and analysis.

Stochastic optical reconstruction microscopy (STORM) imaging was performed as described recently (29) using anti-H2A (Abcam; catalogue number ab88770, 1:150) and rabbit anti-γ-H2A.X (S^P^Q) (Cell Signaling; catalogue number 9718S, 1:300) as primary antibodies. Alexa Schematron Fluor 594 goat anti-rabbit antibody (Life Technologies; 1:500) was used as a secondary antibody. Parasite nuclei were labeled with YOYO-1 (1:300; Life Technologies) for orientation and were not subjected to STORM. STORM was performed with a Nikon Eclipse Ti-E microscope with a CFI Apo total internal-reflection fluorescence (TIRF) × 100 differential inference contrast (DIC) N2 oil objective (NA, 1.49; WD, 0.12 mm) as described (29). For each acquisition, 10,000 frames were recorded onto a 256- by 256-pixel region (pixel size, 160 nm) of an Andor iXon-897 electron-multiplying CCD (EMCCD) camera. Superresolution images were reconstructed from a series of the least 5,000 images per channel using the N-STORM analysis module, version 1.1.21, of NIS Elements AR v. 4.40 (Laboratory imaging s.r.o.).

### Total-histone extraction.

Total histones were extracted using an acid extraction method as described previously, with minor modifications (30). All steps were performed at 4°C in buffers containing protease and phosphatase inhibitors to protect the enzymatic interference with posttranslational modifications (PTMs). In brief, 200 ml of parasite cultures (∼10% parasitemia) were saponin lysed and washed with PBS containing protease and phosphatase inhibitors. To prepare the intact nuclei, the cell pellet was resuspended in 1 ml of lysis buffer (20 mM HEPES [pH 7.8], 10 mM KCl, 1 mM EDTA, 1% Triton X-100, and 1 mM dithiothreitol [DTT]) and incubated for 30 min on rotator at 4°C. Following cell lysis, the intact nuclei were washed and pelleted by centrifugation at 10,000 × *g* for 10 min at 4°C. The nuclei were resuspended in 400 μl of 0.4 N H_2_SO_4_ or 0.25 N HCl. The nuclei were incubated on a rotator overnight, and supernatant containing the acid soluble histone fraction was collected after centrifugation at 16,000 × *g* for 10 min.

### Immunoprecipitation.

Immunoprecipitation of PfH2A was performed as described previously (23), with slight modification. In brief, 200 ml of parasite cultures (∼10% parasitemia) were saponin lysed and washed with PBS containing protease and phosphatase inhibitors. Subsequently, the parasite pellet was dissolved in chilled lysis buffer containing 50 mM Tris-HCl (pH 7.5), 150 mM NaCl, 1 mM EDTA, 0.01% SDS, and 1% NP-40 supplemented with protease and phosphatase inhibitors (Roche) and sonicated for 4 to 8 cycles of 10 to 15 s at 45% output using Hielscher UP200S sonicator. The sonicated pellet was incubated for 30 min on ice. The lysate was purified by a few rounds of centrifugation at 10,000 × *g* for 10 min and incubated with primary antibody (anti-H2A antibody), (catalogue number ab88770; Abcam) for 10 to 12 h at 4°C with continuous swirling. The supernatant was further incubated for 4 to 6 h with protein A/G agarose beads (Pierce) at 4°C, and beads were pelleted by centrifugation at 4°C. Beads were then washed with ice-chilled washing buffer. Immunoprecipitated proteins were eluted with SDS Laemmli buffer and used for detection by SDS-PAGE and Western blot analysis.

### Mass spectrophotometry (LC-MS/MS analysis).

To identify the phosphorylated serine of PfH2A, the extracted histones were digested by trypsin and analyzed by liquid chromatography-tandem mass spectrometry (LC-MS/MS) on Q Exactive plus (Thermo). The peptides were identified by Discoverer software version 1.4 against the *Plasmodium* NCBI-NR database and against decoy databases to determine the false-discovery rate (FDR) using the sequest and mascot search engines. Semiquantitation was done by calculating the peak area of each peptide. The area of the protein is the average of the three most intense peptides from each protein. The results were filtered for proteins identified with at least 2 peptides with 1% FDR.
