# Supplementary figures and images for: Phosphorylation of the Canonical Histone H2A Marks Foci of Damaged DNA in Malaria Parasites
Source: mSphere. 2021 Jan 13;6(1):e01131-20. doi: 10.1128/mSphere.01131-20 (PMC7845613; doi:10.1128/mSphere.01131-20)

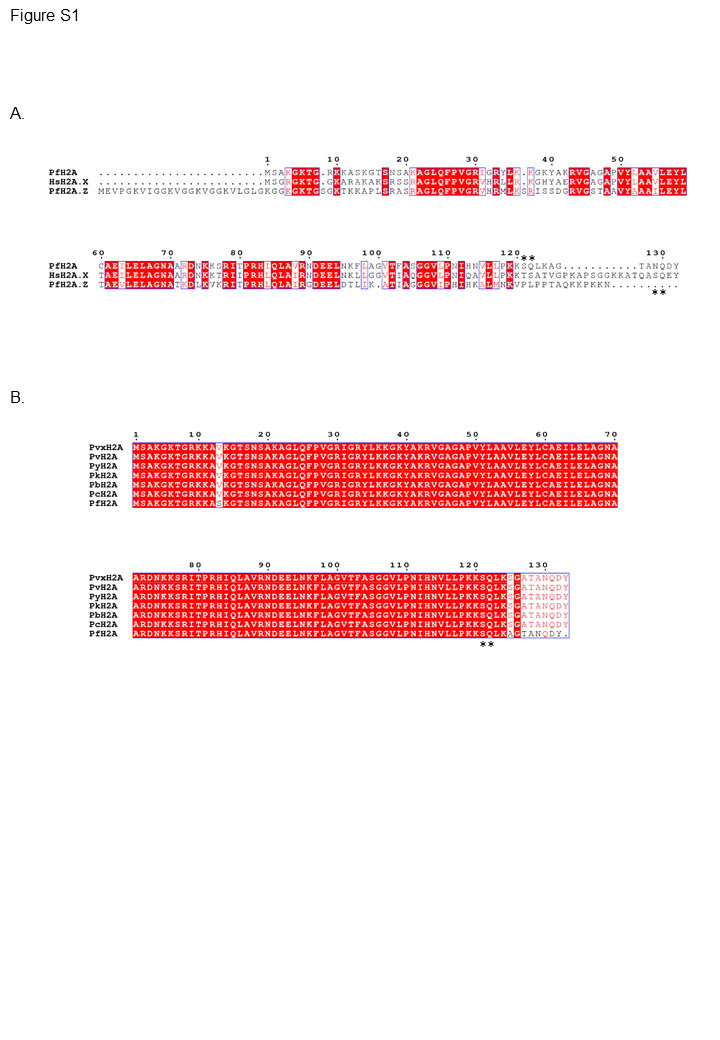

Supplement: FIG S1 [file mSphere.01131-20_sf001.tif]

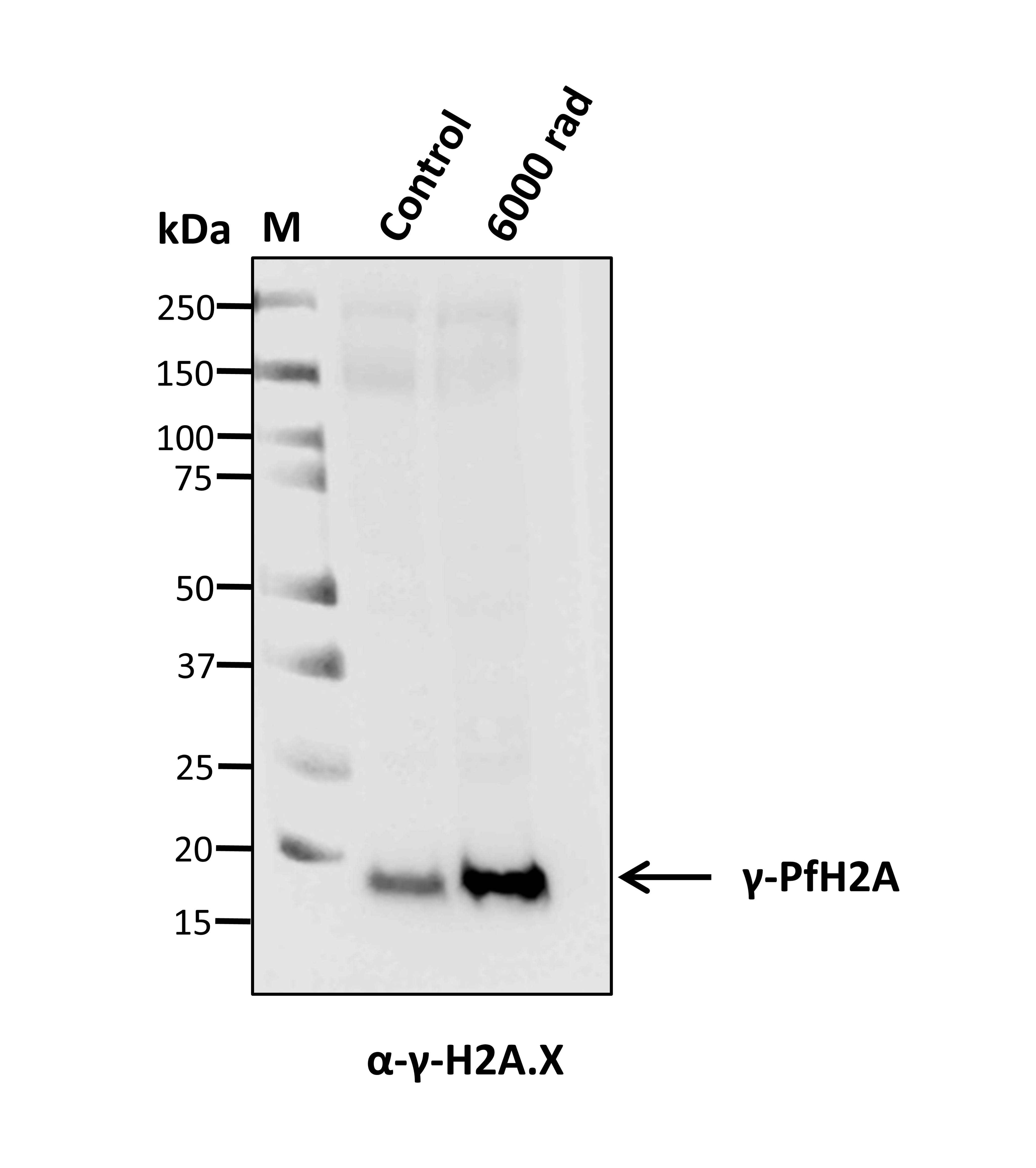

Supplement: FIG S2 [file mSphere.01131-20_sf002.tif]
